# Prediction Model for Identifying Computational Phenotypes of Children with Cerebral Palsy Needing Neurotoxin Treatments

**DOI:** 10.3390/toxins15010020

**Published:** 2022-12-28

**Authors:** Carlo M. Bertoncelli, Michal Latalski, Domenico Bertoncelli, Sikha Bagui, Subhash C. Bagui, Dechelle Gautier, Federico Solla

**Affiliations:** 1 Department of Computer Science, Hal Marcus College of Science & Engineering, University of West Florida, Pensacola, FL 32514, USA; 2 EEAP H Germain and Department of Pediatric Orthopaedic Surgery, Lenval Foundation, University Pediatric Hospital of Nice, 06000 Nice, France; 3 Department of Information Engineering Computer Science and Mathematics, Computer Science and Mathematics, University of L’Aquila, 67100 L’Aquila, Italy; 4 Children Orthopaedic Department, Medical University, 20-059 Lublin, Poland

**Keywords:** prediction model, neurotoxin treatment, cerebral palsy

## Abstract

Factors associated with neurotoxin treatments in children with cerebral palsy (CP) are poorly studied. We developed and externally validated a prediction model to identify the prognostic phenotype of children with CP who require neurotoxin injections. We conducted a longitudinal, international, multicenter, double-blind descriptive study of 165 children with CP (mean age 16.5 ± 1.2 years, range 12–18 years) with and without neurotoxin treatments. We collected functional and clinical data from 2005 to 2020, entered them into the BTX-PredictMed machine-learning model, and followed the guidelines, “Transparent Reporting of a Multivariable Prediction Model for Individual Prognosis or Diagnosis”. In the univariate analysis, neuromuscular scoliosis (*p* = 0.0014), equines foot (*p* < 0.001) and type of etiology (prenatal > peri/postnatal causes, *p* = 0.05) were linked with neurotoxin treatments. In the multivariate analysis, upper limbs (*p* < 0.001) and trunk muscle tone disorders (*p* = 0.02), the presence of spasticity (*p* = 0.01), dystonia (*p* = 0.004), and hip dysplasia (*p* = 0.005) were strongly associated with neurotoxin injections; and the average accuracy, sensitivity, and specificity was 75%. These results have helped us identify, with good accuracy, the clinical features of prognostic phenotypes of subjects likely to require neurotoxin injections.

## 1. Introduction

Cerebral palsy (CP) comprises a group of non-progressive motor control and posture disorders due to brain damage during the early stages of development. Clinical manifestations include involuntary movements or gait abnormalities, movement alterations (loss of tone or spasticity of the trunk and limbs with exaggerated reflexes), and abnormal posture [1]. The progression of dynamic contracture into fixed contracture is an issue of paramount importance for the effective use of botulinum toxins.

Muscle hyperactivity can be effectively reduced by injecting botulinum toxins [2]. Over the past 25 years, botulinum toxins have emerged as the most widely used medical intervention in children with CP. Botulinum toxins reduce muscle strength and tone, with a small, short-term improvement in walking and function. The lack of knowledge on pathophysiology and mechanisms leading from hypertonia to contractures explains the complexity of CP. In addition, little is known about the most commonly used treatment, botulinum toxin (BTX) [2].

Electronic Medical Record (EMR) data are very useful in identifying health outcomes. They contain rich clinical information, including laboratory test results, vital signs, discharge summaries, progress notes, and radiologic and pathologic images and reports, among other information.

Computational phenotyping is the creation of computer-processable algorithms to identify individuals with specific health conditions, diseases, or clinical events from EMR data [3,4].

Large-scale EMRs are an obvious data source for clinical phenotype discovery research. However, EMRs are designed primarily for clinical care, and some effort is required to adapt them as a data source for research. EMRs have been employed in clinical [5,6] and genomic [7,8] research using expert domain knowledge to devise phenotype specifications that identify clinical cohorts of interest manually.

One of the key changes needed to achieve precision and personalized medicine is to let the data speak for themselves, to tell us what the phenotypes are, abandoning the use of historical clinical descriptions of each disease. This view is supported in recent studies, indicating that long-recognized diseases such as spasticity in CP are not single entities but collections of many different phenotypes that may or may not coincide with historical disease boundaries [2].

The analysis of EMR with machine-learning methods could help to clarify these issues.

Machine learning (ML) is a contemporary artificial intelligence discipline for analyzing complex data. ML employs algorithms to find patterns in data that are not obvious to humans. Regression and logistic regression (LR) are among the first supervised ML algorithms for creating predictive health models [9,10,11]. ML algorithms are considered supervised if the output classes are labeled (e.g., BTX treatment, yes/no). Thus, supervised ML prediction models can help identify patients who will undergo BTX treatment.

Prediction models can predict the probability (Prob) of a condition (e.g., BTX treatment) being present [12,13,14]. In supervised ML algorithms, the output is obtained from labeled training samples. Through the training examples, the program learns a function (e.g., logistic regression) that will predict new incoming patients with unknown conditions.

In previous studies, we developed [1] and validated [15] “PredictMed,” a supervised ML model to predict neuromuscular scoliosis and hip dysplasia [10,16], gastrostomy placement [17], and identify factors associated with intellectual disability [18] and autism spectrum disorder [19] in individuals with CP. PredictMed has also proven effective in predicting osteoarthritis in young adults using statistical data mining and machine learning [9].

In the present study, we implemented and externally validated the BTX-PredictMed ML model to predict prognostic phenotypes of children with CP needing BTX treatment.

Following the development of a prediction model, external validation is strongly recommended, that is, to evaluate the model’s performance on other participant data not used for model development [13,20,21,22]. External validation requires that predictions about outcomes be made using the original model for each subject in the new dataset, and be compared with observed outcomes [23]. In this study, we applied the same predictive model to patients from different centers and countries to evaluate the model’s performance through external validation.

From a clinical perspective, a reliable predictive model to identify the phenotype of children with CP who need BTX treatment would allow healthcare providers to recruit and schedule patients more efficiently, thereby reducing medical costs.

From a research perspective, this validated predictive model is easily adaptable and could be used in different fields of medicine.

## 2. Results

We developed and externally validated BTX-PredictMed, a statistical machine-learning model to identify factors associated with neurotoxin treatments for children with CP from two European centers. 

This is the first study highlighting the influence of neuromuscular scoliosis, truncal tone disorders, and type of etiology as features constituting the prognostic phenotypes of CP children needing BTX treatment. This study also confirmed previous results [2,24,25,26,27,28,29] about spasticity, equines foot, hip dysplasia, and manual ability as clinical features predicting the need for BTX treatment for this population.

In univariate analysis, the factors linked with neurotoxins treatments were:

- Neuromuscular scoliosis: *p* = 0.0013, Odds ratio (OR) = 2.7;
- Equines foot: *p* < 0.001, OR = 4.1;
- Type of etiology: prenatal > peri/postnatal causes, *p* = 0.05, OR = 0.53.

Factors linked with neurotoxins treatments in multivariate analysis were:

- Upper limbs ability, *p* < 0.001, OR = 3;
- Trunk muscle tone disorders, *p* = 0.02, OR = 1.9;
- The presence of spasticity, *p* = 0.01, OR = 2;
- Dystonia, *p* = 0.004, OR = 5.3;
- Hip dysplasia, *p* = 0.005, OR = 4.

The multivariate analysis had an accuracy of 76%, sensitivity of 67%, specificity of 81%, and an average of 75%.

## 3. Discussion

The present study has shown that children with CP with equines foot, hip dysplasia, and dystonia were four to five times more likely to undergo neurotoxin treatments compared to a similar group that lacked these clinical features. Low levels of MACS (OR = 3), the presence of spasticity (OR = 2), neuromuscular scoliosis (OR = 2.9), and truncal tone disorders (OR = 1.9), are also strong predictors of phenotypes of CP children needing BTX treatment.

On the opposite side, prenatal etiology appears to be less related to BTX injections than perinatal or postnatal (OR < 1).

As expected, spastic foot is the prognostic phenotype’s main feature.

Since the nineties, botulinum treatment of spastic equines foot has been recommended [30,31,32]. In young children with CP and high GMFCS, multilevel injection of BTX can be used for focal treatment of spasticity, particularly for the lower extremity [30].

Injection and distal injection were significantly related to a more significant gain in gross motor function in the younger age group [30]. The functional hindrance of the spastic equines foot is often the initial obstacle noted by parents and specialists. 

BTX injections into the gastrocnemius and soleus muscles have been effective regardless of the duration of treatment and the number of sessions. However, cerebral palsy, the patient’s age, and the impairment level [33] influence this efficacy. In CP children with upper and lower limb spasticity, there is increasing evidence of the time-limited beneficial effect of BTX in decreasing muscle tone. Decreased muscle tone in the lower limbs may translate into improved ambulation in children with CP with spastic equino-varus [30].

We have noticed that children with CP and lower MACS scores (better manual skills) will be more likely to undergo neurotoxin treatment. We, therefore, assume that casts, orthoses and/or orthopedic surgery, should be preferred in case of severe joint deformity related to strong, long-lasting spasticity [33].

Despite the association of BTX and hip dysplasia being classic and widely recommended in the literature [28,29,34], high-quality evidence of the prevention of hip displacement is lacking. From a physiopathologic mechanism perspective, BTX treatment is recommended [27] in patients with initial subluxation or with strong spasticity and concomitant risk factors of dislocation before radiologic evidence of subluxation [16,17]; the high odds ratio confirms this trend. 

We noted that dystonia was strongly associated with BTX treatment, even if this practice is sparsely recommended in the literature [27]. Unfortunately, our data do not allow increasing evidence pro or contra to this indication. We plan further research on this topic in the future.

There is a pronounced trend toward BTX treatment in CP children with spasticity [2,25,26,27,28,29]. The present study specifies the phenotype subtypes: about half of the patients with diplegia received BTX, and roughly one-third of patients with hemiplegia, triplegia, or quadriplegia.

Despite this, we have highlighted the link between BTX treatments, neuromuscular scoliosis, and truncal tone disorder. BTX is poorly described for neuromuscular scoliosis treatment [25], and data support its inefficiency [26]. With regard to truncal tone disorder, this subject is nearly absent from the literature. 

According to the present research, prenatal etiology appears to be less associated with BTX than perinatal or postnatal. Since the prediction model scored a low odds ratio and there are no studies on this, future research is needed to confirm this finding.

Patients with focal/segmental disabling spasticity are ideal candidates for BTX treatment, and there is a growing need for the early selection of suitable candidates [35]. An algorithm has been implemented to support managing adult patients with disabling spasticity by aiding patient selection for BTX treatments [35]. Identifying the prognostic phenotype through BTX-PredictMed could be a reliable support for identifying and selecting younger patients.

Similar results in two separate groups and the totality of patients confirm the validity of the prediction model, which can also be applied to other fields of medical research.

The availability of a robust predictive algorithm would allow healthcare providers to better understand, prevent and manage orthopedic deformations during growth by delaying or avoiding fixed contractures. It would also facilitate early consultation with a qualified neurotoxin specialist, improving the overall quality of care for patients and families. The personalization of therapies would also lead to a reduction in costs.

### 3.1. Tolerance and Precautions

Side effects were rare and mostly transient: cramps, pain, or hematoma at the injection site, rashes, and pseudo-influenza syndromes. We have not noted any contraindications, such as amyotrophic lateral sclerosis or major respiratory occlusion. Other problems, such as myasthenia gravis, Lambert-Eaton syndrome, or amyotrophic lateral sclerosis, have not been found either. Adherence to good practice recommendations, such as adherence to low doses in the first injection and delays between injections, remains the best prevention [24].

### 3.2. Limitations

Limitations of this study include the limited number of patients and the retrospective analysis. This resulted in high specificity and accuracy of diagnosis, while sensitivity was moderate.

The study of the predictive performance of the algorithm by increasing the number of patients and independent variables (>15) will be our next issue.

For the current study, we had a limited cohort of patients to study (hundreds). We plan to study and fine-tune the model on a much larger number (thousands) to confirm and possibly improve the model’s predictive performance. In this regard, PredictMed has already achieved excellent results in predicting osteoarthritis in adults using statistical data mining and machine learning [9]. At this stage, we plan to calibrate this model on a much larger database to test its potential overfitting (e.g., by studying a receiver operating characteristic curve) due to the limited number of patients and with respect to a large number of features and independent variables [10]. We also plan to use Lasso (L1) and Ridge (L2) regularization techniques to improve PredictMed model and implement clinical decision support systems, a tool supporting professionals in making medical decisions.

## 4. Materials and Methods

### 4.1. Study Design

This longitudinal, multicenter, multinational study was conducted between June 2005 and June 2021. For model implementation, data collection and assessments were conducted in the last six months of 2017, while data analysis began in June 2018 and lasted 24 months.

External validation followed the guidelines of the “Transparent Reporting of a multivariable prediction model for Individual Prognosis or Diagnosis” (TRIPOD) Statement [13,14,15].

We compared two groups of CP children with and without BTX treatment in a double-blind study. These CP children, treated in specialized units, had severe motor disorders and cognitive impairment. The development data showed no differences in setting, eligibility criteria, outcome, and predictors.

**Figure 1:**
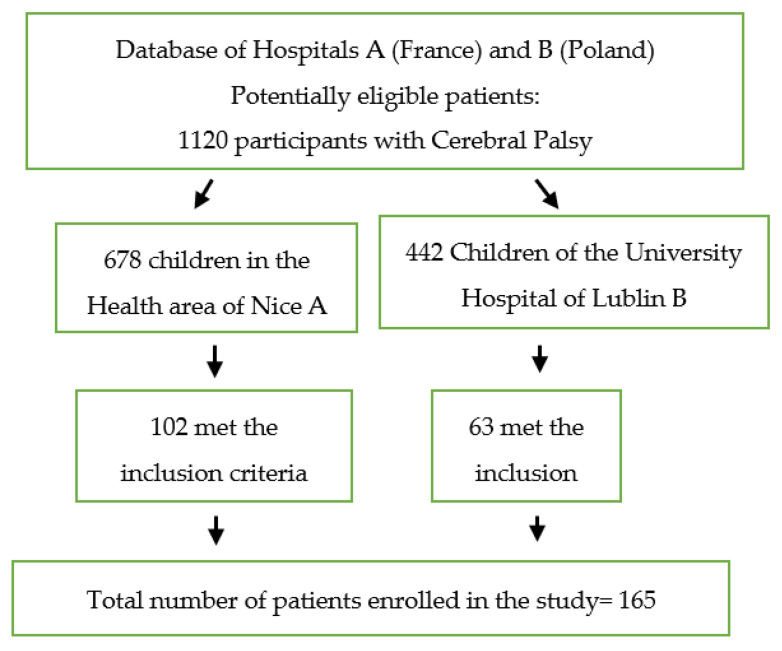
Flow diagram of study participants for analysis.

The flow diagram of study participants for analysis is shown in Figure 1. All consented to be enrolled in the study (Figure 1).

The mean age was 15.7 years (range: 12–18 years; standard deviation [SD] 1.8)**.** The mean follow-up was 5.1 years (range: 3–12 years). 165 patients (91 male, 74 female) assessed between June 2005 and June 2020 were included (Table 1). There were no dropouts during the trial period.

### 4.2. Botulin Toxin Clinical Use

BTX treatment specialists selected children requiring BTX injections based on their clinical and functional characteristics. Once information on possible side effects was provided, the patient’s and/or parents’ approval was explicitly expressed. The date of injection, doses and muscles treated, and pain assessment through a visual analog scale, were recorded.

The specialties Dysport, Botox, Neurobloc, and Xeomin, were available and used for our patient’s neurological conditions. We determined the location of the muscles by palpation, ultrasound, or electromyogram (EMG) needle with injection. This allowed for the noninvasive identification of muscles and surrounding structures [25], and these procedures are especially beneficial in children.

### 4.3. The Doses

The dose depends on the patient’s weight, the severity of the spasticity, the number of muscles treated, the type of toxin, and the size of the muscle. The units were different and were not international units—there is no recognized equivalence: 1 mL for Botox, 2.5 mL for Dysport, 100 Allergan units/mL, and 100 Speywood units per 1 to 2.5 mL. The maximum recommended total dose for children was:

- For Botox, 300 units per session and 20 Allergan units/kg;
- For Dysport, 1000 units per session and 30 Speywood units/kg (professional agreement).

In adults, the recommended total dose is:

- For Botox, 500 Allergan units;
- For Dysport, 1500 Speywood units.

In the case of the first injection, lower initial doses were recommended, especially in patients with comorbidities:

- For Botox, 3 to 8 units/kg without exceeding 300 units per session;
- For Dysport, 10 units/kg in unilateral injections and 20 units/kg in bilateral injections without exceeding 1000 units per session.

We envisaged at least three months’ break between two sessions [24].

### 4.4. Measurements

All data were collected from the Electronic Medical Records by the senior author. Medical notes were written by a multidisciplinary team that included pediatric neurologists, epidemiologists, pediatricians, orthopedic surgeons, and physiotherapists. Narrative notes were coded and filled in the database of BTX-PredictMed [9,20].

Data on diagnosis, etiology, type of spasticity, functional assessments, epilepsy, clinical history, and radiology were collected anonymously between 2005 and 2020.

CP etiology was classified as [1]:

- antenatal: cerebral malformation, genetic, prematurity, infection, vascular;
- perinatal: anoxic, infectious ischemic;
- postnatal: postnatal anoxic/ischemic injury epilepsy, cranial trauma, infectious.

**Figure 2:**
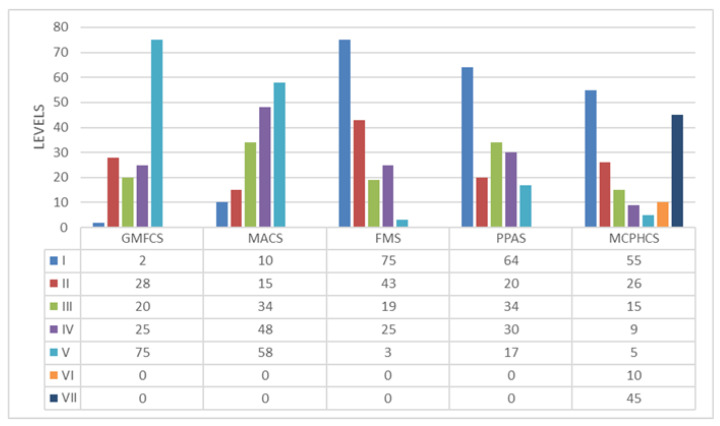
Distribution of patients according to the Manual Ability Classification System (MACS), Gross Motor Function Classification System (GMFCS), Melbourne Cerebral Palsy Hip Classification Scale (MCPHCS), Functional Mobility Scale (FMS), and Posture and Postural Ability Scale (PPAS). Nonapplicable (0).

Motor function was assessed using the Gross Motor Function Classification System (GMFCS) and the Manual Ability Classification System (MACS) [15,24]. Both have a 5-point classification system with higher scores indicating worse motor functioning (Figure 2).

Scoliosis was defined by a Cobb angle > 10° on the spinal radiograph and classified as severe at a Cobb angle > 40° [1,15] (Table 1).

Neurological status was assessed by the presence of hypertonia in the upper or lower extremities, the type of spastic disorder (hemiplegia, diplegia, tri/quadriplegia), the severity of epilepsy, and the presence of dystonia. The modified Ashworth Scale of Bohannon and Smith and the modified Tardieu Scale [1,15] have been used to quantify spasticity.

The severity of epilepsy has been determined by pediatric epileptologists and classified as “well-controlled” or “intractable” [36] based on the guidelines of the International League Against Epilepsy. These guidelines define intractable epilepsy as a continuous seizure despite treatment attempts with at least two antiepileptic drugs [37,38] (Table 1).

Dysplasia was estimated based on the Perkins line: 0% was assigned if the migration percentage (MP) was negative and the lateral margin of the femoral head was medial to the Perkins line. Percent migration (MP) was scored as 100% when the entire femoral head was lateral to the Perkins line.

The hips were classified as normal (MP less than 33%), subluxated (MP = 33 up to 89%) or luxated (MP ≥ 90%) according to the migration percentage [16,17]. Clinical measurement of the hip focused on internal rotation and hip abduction. The modified Harris score (MHHS) [16] was used to assess hip function, pain, and gait. The Melbourne Cerebral Palsy Hip Classification Scale (MCPHCS) [39,40] was used to classify hip morphology. In the case of multiple radiographs, a pediatric orthopedic surgeon evaluated the most recent one. All patients had at least one pelvic radiograph.

Trunk functional abilities were ascertained with the Functional Mobility Scale (FMS) [16], the Posture and Postural Ability Scale (PPAS), and the Lower Extremity Functional Scale (LEFS) [17]. Trunk muscle tone was assessed with the Trunk Impairment Scale (TIS) [16] (Figure 2).

The variables investigated were:

o Neurotoxins treatments (NT);
o Presence of Neuromuscular scoliosis (NS);
o Trunk muscle tone disorder (TT);
o Spasticity (SP);
o Dystonia (D);
o Epilepsy (E);
o Hip Dysplasia (HD);
o Equines foot (EF);
o Gastrostomy feeding (GA);
o Sex (SE);
o Etiology (ET);
o GMFCS;
o MACS.

ET, TT, SP, D, SE, GMFCS, MACS, and E were assessed in the first control; NT, NS, HP, GA, and EF were assessed in the last control.

### 4.5. Statistical Analysis

We performed Fisher’s exact tests and developed contingency tables [41] for identifying the distribution frequencies and the confidence intervals of factors associated with BTX treatments. Then, we used the web-based epidemiological calculators MedCalc^®^ statistical software 20.123 and OpenEpi software 3.01 [42,43] to calculate 95% confidence intervals, odds ratios, and *Z*-statistics (Table 2).

The glm() function of the open-source software R 4.2.2 [44,45,46] was used to predict each patient’s probability of undergoing BTX treatment; the common thresholds for selecting relevant variables (with *p*-value < 0.2) [44,45] were employed as independent input variables in a bespoke multiple logistic regression model [44,46]. The binary dependent variable was the presence of BTX treatment (yes/no).

The selected Independent variables entered in BTX-PredictMed were: ET, TT, SP, D, E, NS, GMFCS, SE, MACS, and HD.

In accordance with the statistical learning theory reported by Vapnik and Chervonen-kis [47], we divided the patients into a “training set” to train the LR model and a “test set” to check the performance of the model. We checked whether each subject in the “test set” was correctly predicted as a potential developer of epilepsy (or not) by calculating the sensitivity, specificity, and accuracy [43].

To minimize the dependence on the composition of the training and test sets, cross-validation was used. Cross-validation is a technique to evaluate the generalization of the results of a statistical analysis on an independent data set. We randomly generated 20 couples of training and test sets; for each couple, we calculated the sensitivity, specificity, and accuracy of the predictions; and finally averaged all couples. Accuracy, sensitivity, and specificity were defined in terms of True Positive (TP), True Negative (TN), False Negative (FN), and False Positive (FP) [43].

Then, we compared the predictions with the patient’s known status (e.g., whether he/she has BTX or not) for each patient in the “test set,” calculating the sensitivity, specificity, and accuracy of the predictive logistic regression algorithm (Table 3).

## Figures and Tables

**Table 1:** Clinical presentation according to the presence or absence of Neurotoxin Treatments.

Patients Profile	Pediatric Hospital A			Children Hospital B			Multicenter A + B
	Neurotoxin Treatments			Neurotoxin Treatments			Total (%)
	Yes (%)	No (%)	Total (%)	Yes (%)	No (%)	Total (%)	
Patients n. (%)	17 (17)	85 (83)	102 (100)	49 (77)	14 (23)	63 (100)	165 (100)
Male	35 (58)	25 (42)	60 (100)	22 (70)	9 (30)	31 (100)	91 (55)
Female	23 (55)	19 (45)	42 (100)	18 (58)	14 (42)	32 (100)	74 (45)
Average age (mean, SD)	16.4 (1.8)	16.8 (1.8)	16.6 (1.8)	15.8 (1.8)	16.0 (1.8)	15.9 (1.8)	16.2 (1.8)
Spasticity, n. (%)	16 (21)	59 (79)	75 (100)	34 (48)	20 (52)	54 (100)	129 (78)
Hemiplegia	2 (22)	7 (78)	9 (100)	3 (75)	1 (25)	4 (100)	13 (8)
Diplegia	1 (6)	15 (94)	16 (100)	20 (86)	3 (14)	23 (100)	39 (24)
Tri/quadriplegia	13 (26)	37 (74)	50 (100)	11 (41)	16 (59)	27 (100)	77 (68)
Dystonia n. (%)	10 (71)	4 (29)	14 (100)	8 (66)	4 (36)	12 (100)	26 (16)
Well-controlled Epilepsy, n. (%)	10 (20)	40 (80)	50 (100)	23 (79)	6 (21)	29 (100)	79 (48)
Intractable Epilepsy	4 (18)	18 (82)	22 (100)	10 (77)	3 (23)	13 (100)	35 (20)
No Epilepsy	3 (10)	27 (90)	30 (100)	16 (76)	5 (24)	21 (100)	51 (31)
Severe Scoliosis (%)	23 (59)	16 (41)	39 (100)	16 (53)	14 (47)	30 (100)	69 (41)
Equines Foot (%)	31 (75)	10 (25)	41 (100)	21 (75)	7 (25)	28 (100)	69 (41)
Hip Dysplasia (%)	18 (56)	14 (44)	32 (100)	13 (59)	9 (41)	22 (100)	54 (38)
Truncal tone disorder (%)	11 (21)	42 (79)	53 (100)	29 (74)	10 (26)	39 (100)	92 (56)
Ante-natal Causes	10 (16)	54 (84)	64 (100)	21 (84)	4 (16)	25 (100)	89 (54)
Perinatal Causes	4 (14)	25 (86)	29 (100)	24 (72)	9 (28)	33 (100)	62 (37)
Postnatal Causes	3 (34)	6 (66)	9 (100)	4 (80)	1 (20)	5 (100)	14 (9)

**Table 2:** Contingency table comparing subjects with and without Neurotoxin Treatments using the Fisher’s exact test.

Independent Variables		Multicenter Pediatric Hospital A + Children Hospital B						Hospitals	
								A	B
		Neurotoxin Treatments		Odds Ratio	95% CIs	Z Statistic	*p* Value	*p* Value	*p* Value
		Yes	No						
Neuromuscular Scoliosis (NS)	Yes	39	30	2.86	1.50–5.43	3.20	0.0013	0.007	0.006
	No	30	66						
Equines Foot (EF)	Yes	45	30	4.12	2.13–7.95	4.22	<0.0001	<0.0001	<0.0001
	No	24	66						
Etiology (ET) PreNatal > Peri/PostNatal causes	Yes	31	58	0.53	0.28–0.99	1.96	0.05	0.05	0.05
	No	38	38						

**Table 3:** List of the logistic regression coefficients associated with the presence of Neurotoxins Treatments.

Logistic Regressions					
Independent Variables	Odds Ratio		Standard Error	Z Ratio	Prob(>|Z|) *p* Value
	Logarithm	Linear			
Intercept	1.563	4.77	0.879	1.777	0.075
Scoliosis (NS)	0.146	0.863	0.476	−0.308	0.757
Truncal Tone Disorder (TT)	0.626	1.870	0.277	2.258	0.023
Etiology	0.077	1.080	0.316	0.246	0.805
Spasticity (SP)	0.677	1.967	0.285	2.374	0.017
Dystonia (D)	1.670	5.312	0.583	2.864	0.004
Epilepsy (E)	0.227	1.254	0.349	0.649	0.515
Gender (SE)	0.512	1.668	0.421	1.215	0.224
GMFCS score	0.299	0.741	0.312	−0.957	0.338
MACS score	1.085	2.959	0.250	−4.334	<0.001
Hip Dysplasia (HD)	1.392	4.022	0.500	2.7822	0.05

Logistic Regression: The increasing of TT, SP (Quadriplegia/triplegia >Diplegia> hemiplegia), D, MACS score, and HD are factors associated with the presence of Neurotoxin Treatments (in the “Odds Ratio-Linear” column). This means, more precisely, that for every unit increase in SP, the log odds = ln(p/1−p) increases 1.967 times (where *p* = probability of having Neurotoxins Treatments). The “Prob(>|z|)” column indicates the significant strength of the respective parameter in terms of the *p*-value as the presence of Neurotoxin Treatments. This means that the significance of TT, SP, D, MACS score, and HD in predicting the presence of Neurotoxin Treatments is very probable, with a *p*-value < 0.05. The best machine learning model score performed with an accuracy of 76%, sensitivity of 67%, specificity of 81%, and an average score of 75%.

## Data Availability

Not applicable.

## References

[1] Bertoncelli C.M., Solla F., Loughenbury P.R., Tsirikos A.I., Bertoncelli D., Rampal V. (2017). Risk factors for developing scoliosis in cerebral palsy: A cross sectional descriptive study. J. Child Neurol..

[2] Multani I., Manji J., Hastings-Ison T., Khot A., Graham K. (2019). Botulinum Toxin in the Management of Children with Cerebral Palsy. Pediatr. Drugs.

[3] Lasko T.A., Denny J., Levy M.A. (2013). Computational Phenotype Discovery Using Unsupervised Feature Learning over Noisy, Sparse, and Irregular Clinical Data. PLoS ONE.

[4] Seymour T., Frantsvog D.A., Graeber T. (2012). Electronic Health Records (EHR). Am. J. Health Sci..

[5] Denny J.C., Spickard A., Johnson K.B., Peterson N.B., Peterson J.F., Miller R.A. (2009). Evaluation of a Method to Identify and Categorize Section Headers in Clinical Documents. J. Am. Med. Inform. Assoc..

[6] Dean B.B., Lam J., Natoli J.L., Butler Q., Aguilar D., Nordyke R.J. (2009). Use of electronic medical records for health outcomes research: A literature review. Med. Care Res. Rev..

[7] Kho A.N., Pacheco J.A., Peissig P.L., Rasmussen L., Newton K.M., Weston N., Crane P.K., Pathak J., Chute C.G., Bielinski S.J. (2011). Electronic Medical Records for Genetic Research: Results of the eMERGE Consortium. Sci. Transl. Med..

[8] Kohane I.S. (2011). Using electronic health records to drive discovery in disease genomics. Nat. Rev. Genet..

[9] Bertoncelli C.M., Altamura P., Bagui S., Vieira E.R., Costantini S., Monticone M., Solla F., Bertoncelli D. (2022). Predicting osteoarthritis in adults using statistical data mining and machine learning. Ther. Adv. Musculoskelet. Dis..

[10] Bertoncelli C.M., Altamura P., Bertoncelli D., Rampal V., Vieira E.R., Solla F. (2020). PredictMed: A Machine Learning Model for Identifying Risk Factors of Neuromuscular Hip Dysplasia: A Multicenter Descriptive Study. Neuropediatrics.

[11] Bertoncelli C.M., Solla F. (2020). Machine learning for monitoring and evaluating physical activity in cerebral palsy. Dev. Med. Child Neurol..

[12] Hastie T., Tibshirani R., Friedman J. (2009). Boosting and additive trees. The Elements of Statistical Learning.

[13] Collins G.S., Reitsma J.B., Altman D.G., Moons K.G.M. (2015). Transparent Reporting of a multivariable prediction model for Individual Prognosis or Diagnosis (TRIPOD): The TRIPOD statement. BMJ.

[14] Pereira F., Mitchell T., Botvinick M. (2009). Machine learning classifiers and fMRI: A tutorialoverview. Neuroimage.

[15] Bertoncelli C.M., Bertoncelli D., Elbaum L., Latalski M., Altamura P., Musoff C., Rampal V., Solla F. (2018). Validation of a Clinical Prediction Model for the Development of Neuromuscular Scoliosis: A Multinational Study. Pediatr. Neurol..

[16] Bertoncelli C.M., Altamura P., Vieira V.R., Bertoncelli D., Solla F. (2020). Predicting hip dysplasia in teenagers with cerebral palsy in order to optimize prevention and rehabilitation. A longitudinal descriptive study. Dev. Neurorehabilit..

[17] Bertoncelli C.M., Altamura P., Vieira E., Bertoncelli D., Latalski M., Berthet S., Solla F. (2020). Predictive Model for Gastrostomy Placement in Adolescents with Developmental Disabilities and Cerebral Palsy. Nutr. Clin. Pr..

[18] Bertoncelli C.M., Altamura P., Vieira E.R., Bertoncelli D., Thummler S., Solla F. (2019). Identifying factors associated with intellectual disabilities in teenagers with cerebral palsy using a predictive learning model. J. Child. Neurol..

[19] Bertoncelli C.M., Altamura P., Vieira E.R., Bertoncelli D., Solla F. (2019). Using Artificial Intelligence to Identify Factors Associated with Autism Spectrum Disorder in Adolescents with Cerebral Palsy. Neuropediatrics.

[20] Bertoncelli C.M., Altamura P., Vieira E.R., Iyengar S.S., Solla F., Bertoncelli D. (2020). PredictMed: A logistic regression-based model to predict health conditions in cerebral palsy. Health Inform. J..

[21] Moons K.G., Altman D.G., Reitsma J.B., Collins G.S. (2015). New guideline for the reporting of studies developing, validating, or updating a multivariable clinical prediction model: The TRIPOD statement. Adv. Anat. Pathol..

[22] Moons K.G.M., Kengne A.P., Grobbee D.E., Royston P., Vergouwe Y., Altman D.G., Woodward M. (2012). Risk prediction models: II. External validation, model updating, and impact assessment. Heart.

[23] Altman D.G., Vergouwe Y., Royston P., Moons K.G. (2009). Prognosis and prognostic research: Validating a prognostic model. BMJ.

[24] Hareb F., Rampal V., Bertoncelli C.M., Rosello O., Solla F. (2020). Botulinum toxin in children with cerebral palsy: An update. Neuropediatrics.

[25] Mirska A., Kułak W., Okurowska-Zawada B., Dmitruk E. (2019). Effectiveness of multiple botulinum toxin sessions and the duration of effects in spasticity therapy in children with cerebral palsy. Childs Nerv Syst..

[26] Barlaan Lukban M., Rosales R.L., Dressler D. (2009). Effectiveness of botulinum toxin A for upper and lower limb spasticity in children with cerebral palsy: A summary of evidence. J. Neural Transm..

[27] Bohn E., Goren K., Switzer L., Falck-Ytter Y., Fehlings D. (2021). Pharmacological and neurosurgical interventions for individuals with cerebral palsy and dystonia: A systematic review update and meta-analysis. Dev. Med. Child Neurol..

[28] Lin C.Y., Chung C.H., Matthews D.J., Chu H.Y., Chen L.C., Yang S.S., Chien W.C. (2021). Long-term effect of botulinum toxin A on the hip and spine in cerebral palsy: A national retrospective cohort study in Taiwan. PLoS ONE.

[29] Young Choi J., Kim S.K., Park E.S. (2019). The Effect of Botulinum Toxin Injections on Gross Motor Function for Lower Limb Spasticity in Children with Cerebral Palsy. Toxins.

[30] Aydil S., Akpinar F.M., Akpinar E., Beng K., Yagmurlu M.F. (2019). Effectiveness of Multilevel Botulinum Toxin a Injection with Integrated Treatment Program on Spasticity Reduction in Non-Ambulatory Young Children with Cerebral Palsy. Med. Princ. Pract..

[31] Mall V., Heinen F., Linder M., Philipsen A., Korinthenberg R. (1997). Treatment of cerebral palsy with botulinum toxin A: Functional benefit and reduction of disability. Three case reports. Pediatr. Rehabil..

[32] Corry I.S., Cosgrove A.P., Duffy C.M., McNeill S., Taylor T.C., Graham H.K. (1998). Botulinum toxin A compared with stretching casts in the treatment of spastic equinus: A randomised prospective trial. J. Pediatr. Orthop..

[33] Chaléat-Valayer E., Parratte B., Colin C., Denis A., Oudin S., Bérard C., Bernard J.C., Bourg V. (2011). A French observational study of botulinum toxin use in the management of children with cerebral palsy: BOTULOSCOPE. Eur. J. Paediatr. Neurol..

[34] Lee Y., Lee S., Jang J., Lim J., Ryu J.S. (2021). Effect of Botulinum Toxin Injection on the Progression of Hip Dislocation in Patients with Spastic Cerebral Palsy: A Pilot Study. Toxins.

[35] Biering-Soerensen B., Stevenson V., Bensmail D., Grabljevec K. (2022). European expert consensus on improving patient selection for the management of disabling spasticity with intrathecal baclofen and/or botulinum toxin type A. J. Rehabil. Med..

[36] Berg A.T. (2006). Defining intractable epilepsy. Adv. Neurol..

[37] Sinha S., Siddiqui K.A. (2011). Definition of intractable epilepsy. Neurosciences.

[38] Berg A.T. (2009). Identification of pharmacoresistant epilepsy. Neurol. Clin..

[39] Terjesen T. (2012). The natural history of hip development in cerebral palsy. Dev. Med. Child Neurol..

[40] Lins L.A., Watkins C.J., Shore B.J. (2019). Natural History of Spastic Hip Disease. Disease J. Pediatr. Orthop..

[41] Solla F., Tran A., Bertoncelli D., Musoff C., Bertoncelli C. (2018). Why a *p*-value is not enough. Clin. Spine Surg..

[42] Sullivan K., Andrew D., Minn Minn S. (2009). OpenEpi: A web-based epidemiologic and statistical calculator for public health. Public Health Rep..

[43] Wen Z., Zeng N., Wang N. Sensitivity, specificity, accuracy, associated confidence interval and ROC analysis with practical SAS^®^ implementations. Proceedings of the NESUG Proceedings: Health Care and Life Sciences.

[44] Robert J.T. (1999). An Introduction to Error Analysis: The Study of Uncertainties in Physical Measurements.

[45] Mickey R.M., Greenland S. (1993). The impact of confounder selection criteria on effect estimation. Am. J. Epidemiol..

[46] Maldonado G., Greenland S. (1993). Simulation study of confounder-selection strategies. Am. J. Epidemiol..

[47] Vapnik V. (2013). The Nature of Statistical Learning Theory.

